# Dual-energy CT: Impact of detecting bone marrow oedema in occult trauma in the Emergency

**DOI:** 10.1093/bjro/tzae025

**Published:** 2024-09-11

**Authors:** Muhammad Israr Ahmad, Lulu Liu, Adnan Sheikh, Savvas Nicolaou

**Affiliations:** Department of Radiology, Univeristy of British Columbia, Vancouver, Canada; Department of Radiology, Univeristy of British Columbia, Vancouver, Canada; Department of Radiology, Univeristy of British Columbia, Vancouver, Canada; Department of Radiology, Univeristy of British Columbia, Vancouver, Canada

**Keywords:** dual-energy CT, Musculoskeletal (MSK) trauma, bone marrow oedema, DECT

## Abstract

Dual-energy computed tomography (DECT) is an advanced imaging technique that acquires data using two distinct X-ray energy spectra, typically at 80 and 140 kVp, to differentiate materials based on their atomic number and electron density. This capability allows for the enhanced visualisation of various pathologies, including bone marrow oedema (BMO), by providing high-resolution images with notable energy spectral separation while maintaining radiation doses comparable to conventional CT. DECT’s ability to create colour-coded virtual non-calcium (VNCa) images has proven particularly valuable in detecting traumatic bone marrow lesions (BMLs) and subtle fractures, offering a reliable alternative or complement to MRI. DECT has emerged as a significant tool in the detection and characterisation of bone marrow pathologies, especially in traumatic injuries. Its ability to generate high-resolution images and distinguish between different tissue types makes it a valuable asset in clinical diagnostics. With its comparable diagnostic accuracy to MRI and the added advantage of reduced examination time and increased availability, DECT represents a promising advancement in the imaging of BMO and related conditions.

## Introduction

### Dual-energy CT

Dual-energy computed tomography (DECT) is an imaging technique that acquires data using 2 different X-ray energy spectra. This allows for the differentiation of materials based on their varying energy-dependent X-ray absorption behaviours.^1^ DECT relies on the differential absorption of X-rays at distinct energy levels, typically at 80 and 140 kVp, to discern between various materials based on their atomic number.^2^ Materials with higher atomic numbers, such as calcium, exhibit greater attenuation differences when imaged with X-rays at different energy levels, owing to the photoelectric effect, in contrast to materials with lower atomic numbers, which show minimal attenuation differences.^3^ DECT therefore provides valuable information for diagnosing various conditions, including bone marrow oedema (BMO) and other acute pathologies.^4^

The capacity to distinguish between materials is thus directly related to their atomic number and electron density.^1^ Through the utilisation of independent tube current modulation, low electronic noise detectors, and tin filtering of higher kilovoltage (kV) tube spectra, DECT generates high-resolution images with notable energy spectral separation while maintaining radiation doses comparable to those of conventional CT.^5^

In DECT, a colour overlay image can be created, where the virtual noncalcium (VNCa) content is superimposed on the grey-scale calcium content.^6^ This enables the fusion of bone marrow attenuation content on the anatomic images, allowing for better visualisation of subtle changes in the underlying attenuation of the bone marrow.^7^

By acquiring images at 2 different energy levels, DECT facilitates the decomposition of the imaged tissues into 3 materials and allows for the creation of colour-coded virtual non-calcium (VNCa) images. This technique has found utility in detecting bone marrow lesions (BMLs), including traumatic BMLs, by enabling the visualisation of these lesions even in the absence of cortical disruption or in the presence of fractures.^8^

### Role of dual-energy in detecting marrow oedema

DECT has proven to be valuable in the assessment of BMO and lesions in various clinical scenarios, particularly in the context of traumatic injuries. It has emerged as a promising tool for the visualisation and characterisation of bone marrow pathologies, offering insights into bone injuries and related conditions.^9–13^

Several studies have demonstrated the diagnostic accuracy of DECT in detecting traumatic BMO in the knee, ankle, vertebral compression fractures, and acute wrist fractures.^14^ The use of DECT and virtual non-calcium techniques has been shown to be effective in evaluating post-traumatic bone bruises and BMO in different settings, providing reliable diagnostic information. Moreover, the technique has been compared with MRI and has shown promising results, indicating its potential as an alternative or complementary imaging modality for assessing BMO.^15–18^

### Bone marrow

Bone marrow, a dynamic and complex organ, is the cancellous tissue located within bone cavities. It consists of 2 types: haematopoietic (red) marrow and fatty (yellow) marrow. Red marrow is composed of roughly 40% fat, 40% water, and 20% protein, whereas yellow marrow contains approximately 80% fat, 15% water, and 5% protein.^19^ It is responsible for the production of blood cells, including red blood cells, white blood cells, and platelets. The bone marrow also contains stem cells, which are important for the body’s ability to repair and replenish cells. The distribution of red and yellow marrow in the bone changes with age, with red marrow gradually converting to yellow marrow. In certain conditions such as anaemia, smoking, or obesity, the relative amount of haematopoiesis in the bone marrow can be dynamically affected, leading to changes in the distribution of red and yellow marrow.^13^^,^^20^

Overall, bone marrow plays a crucial role in the body’s immune system and the production of essential blood components.

### Bone marrow oedema

BMO refers to the accumulation of excess fluid in the bone marrow, which can be caused by various factors such as acute trauma, stress fractures, or other pathological conditions.^10^ It is a diagnostic imaging finding typically observed on MRI. It identifies a region of low signal intensity on T1-weighted images and high signal intensity on fat-saturated T2-weighted images of the bone marrow.^21^ Initially, these areas were believed to indicate occult fractures not visible on conventional radiography, corresponding to water signal intensity on MRI likely due to injury-related oedema; however, later it was shown that these regions may also be related to a mix of haemorrhage, tissue damage, fibrosis/scarring, increased vascularity, and areas of lymphocyte infiltration. These findings suggest that the observed changes could result not only from acute injury but also from a variety of other conditions, including infectious, inflammatory, and/or malignancy.^22^

BMO is more complex than simple fluid infiltration into the bony trabeculae and can be seen in conditions such as occult or mildly displaced fractures, pathologic fractures, metastases, ligamentous injuries, and inflammatory arthritis.^23^ BMO is considered a significant clinical phenomenon that can help disease management and treatment strategies, particularly in conditions such as osteoarthritis. It has been the focus of diagnostic research, and various imaging modalities have been evaluated for their accuracy in detecting and characterising BME.

### Causes of BMO

The causes of BMO can be multifactorial and include the following^24^^,^^25^:

1. Trauma: BMO can occur as a result of traumatic injuries, including occult fractures, pathologic fractures, ligamentous injuries, and inflammatory arthritis. It can also occur due to indirect trauma (eg, bone contusion) and overload-induced subacute injury (eg, stress fracture).
2. Ischaemia: Experimental studies have shown that ischaemia can affect the bone and marrow, leading to the development of BMO.
3. Inflammatory Conditions: BMO can be associated with various infectious and inflammatory conditions, as well as neoplastic processes.
4. Metabolic Disorders: Certain metabolic disorders may contribute to the development of BMO.
5. Other Conditions: BMO can also be seen in the context of conditions such as BMLs, osteochondritis dissecans, and rheumatoid arthritis.

### Standard of detecting marrow oedema

MRI is the standard for imaging and visualising BMO. It is detected as an area of low signal intensity on the T1-weighted sequence and increased signal intensity on the fat-saturated T2-weighted sequence. This technique helps visualise minor alterations in the bone marrow attenuation, facilitating the detection of occult or minimally displaced fractures, pathologic fractures, and other osseous lesions.^26^^,^^27^

It is important to note that DECT has also emerged as a valuable tool for detecting BMO in certain cases, particularly in the emergency department setting when access to MRI is not as readily available. DECT has been found to be effective in the assessment of BMO in various parts of the body, such as the knee, wrist, scaphoid, vertebral column, and calcaneus. It has been shown to have incremental diagnostic value, high diagnostic accuracy, and clinical utility in detecting BMO. Additionally, DECT has been compared to MRI and has shown promising results in the detection of traumatic BMO.^28–31^

## Role of conventional imaging in detecting BMO

### Radiography

The role of radiography in detecting trauma or marrow oedema is limited. Conventional radiography is not sensitive enough to detect subtle changes in bone marrow, such as BMO. While it can show cortical disruption in fractures, it may not be able to demonstrate fractures that predominantly involve trabecular bone. Additionally, it may not be effective in immediately assessing traumatic injuries in the absence of cortical disruption. It is not the preferred modality for evaluating BMO or subtle fractures.^32^^,^^33^

### Conventional CT

The importance of conventional CT in evaluating traumatic injuries is considerable. It offers crucial insights into bone structure and density, which are essential for identifying and characterising traumatic bone lesions.^34^^,^^35^ However, it is important to note that conventional CT has limitations in distinguishing specific soft tissue changes and subtle bone marrow abnormalities compared to more advanced imaging techniques such as DECT and MRI.

Conventional CT plays a significant role in the initial assessment of traumatic injuries and can provide important anatomical details and information about the extent of bone involvement. It is often used as a first-line imaging modality in emergency settings due to its widespread availability and ability to quickly visualise bone fractures and associated soft tissue injuries. Additionally, conventional CT can serve as a complementary tool to DECT and MRI in the comprehensive evaluation of traumatic BMLs, providing essential structural information that contributes to a more complete understanding of the injury.^36^^,^^37^

### Nuclear imaging

Nuclear imaging has a limited role in detecting BMO. There are different techniques that can be used to detect marrow oedema in cases of occult trauma.

PET is a highly sensitive imaging tool that can reveal areas with increased tracer uptake, which can be indicative of bone or cartilage damage. In a study by Marks et al,^38^ bone scintigraphy, a type of nuclear medicine imaging, was able to detect bone injuries, which were later confirmed as subchondral bone damage by MRI in 13 patients. Additionally, PET can provide additional morphologic imaging using 18F-Fluoride PET/MR, which presents BMLs more precisely and provides further diagnostic information at a higher diagnostic certainty.^39^^,^^40^

However, it’s important to note that while PET is highly sensitive for detecting BMLs, it has the drawback of low specificity. Therefore, it is not commonly used as the primary imaging modality for traumatic bone marrow lesions (TBMLs).

A recent research employed technetium 99m (99mTc) hydroxydiphosphonate and exhibited promising results for detecting BMO.^41^ Regions affected by oedema, haemorrhage, and trabecular microfractures display elevated uptake in comparison to unaffected areas. The delayed phase of bone imaging with 99mTc is recognised for its high sensitivity to hyperaemia and increased bone turnover linked with stress injuries, albeit with limited specificity.^42^

### Ultrasound

Ultrasound is beneficial for examining bone and cartilage contours, especially when the surrounding soft tissue is painful and inflamed due to injury. It can also help diagnose occult fractures when trauma history is indistinct but a fracture is clinically suspected or in excluding a fracture as a complement to a normal physical examination when the history is supportive.^43^

In the context of TBMLs, ultrasound can effectively image the cortical bone outline and detect changes in the adjacent soft tissue at a fracture site, utilising the high reflectivity at the cortical bone/peri-osseous soft-tissue interface.^44^ It can also help see the associated features in trauma, for example, haematoma, joint effusion, and soft tissue injuries, including muscle, tendon, or ligamentous tears. Ultrasound can also help in dynamic assessment after the trauma.^45^

### Image post-processing

Advanced post-processing algorithms for DECT images have been developed to extract valuable diagnostic insights, with new material-specific and energy-specific applications significantly enhancing skeletal trauma imaging.^46^^,^^47^

Material-specific techniques like VNCa and collagen mapping reconstruction use a 3-material decomposition algorithm (bone mineral, yellow marrow, and red marrow) to differentiate tissues. This method relies on the distinct photon absorption characteristics of these materials across low- and high-kilovolt peak spectra.^48^ Originally demonstrated by Pache et al^48^ in 2010 to highlight BMO, this technique has since been widely employed to delineate BMO in both axial and appendicular skeletal regions.

By subtracting the calcium matrix from cancellous bone, material-specific techniques can identify BMO using qualitative colour-coded maps and quantitative analysis of low attenuation values.^49^

This technique allows for the creation of calcium and Virtual non-Calcium (VNCa) images, where the increased attenuation of bony trabeculae is removed, enabling direct visualisation of the underlying marrow attenuation. The ability to eliminate trabeculae offers a notable advantage over conventional CT, where high-attenuation trabeculae can obscure the underlying marrow attenuation.

Additionally, a colour overlay image can be generated, integrating colour-coded VNCa content onto greyscale calcium content to merge bone marrow attenuation data onto anatomical images.

Collagen mapping assesses ligaments, tendons, and intervertebral disc integrity via characteristic dual-energy ratio values, employing specific material decomposition and colour-coded reconstruction methods.^50^

Energy-specific applications involve decomposing low- and high-energy data to generate virtual monochromatic images (VMI), which significantly reduce metal artefacts and enhance the evaluation of peri-prosthetic bone and adjacent soft tissues.^51^ These advanced techniques enhance the diagnostic capabilities of DECT imaging, particularly in the context of skeletal trauma assessment.

### Spine injuries

Vertebral fractures are frequently observed in clinical practice, and promptly identifying acute fractures is crucial for initiating appropriate treatment to minimise potentially serious consequences.^52^ CT is the standard of imaging for detecting most fractures by direct visualisation of the vertebral morphology. MRI can be used to provide information on the acute nature of the vertebral injury. This makes it possible to distinguish new fractures from pre-existing osteoporotic fractures commonly seen in older individuals. However, its limited cost-effectiveness benefit and lack of availability make its usage controversial in acute clinical settings.^53^ The emergence of DECT has enhanced the capability of CT to detect BMO by directly visualising BMO through creating VNCa images with diagnostic accuracy comparable to MRI.^48^^,^^54^ In a retrospective study consisting of 528 vertebrae in 49 patients, Kaup et al reported an accuracy improvement from 61% with CT alone to 83% with the addition of VNCa in diagnosing fracture, along with a decrease in the need for MRI referrals from 37% to 87% of patients.^55^ In a different study conducted by Cavallaro et al where 730 vertebrae in 88 patients were evaluated by 5 readers, the diagnostic confidence regarding BMO presence was similar with DECT VNCa and MRI. They also reported a cut-off value of −0.43 Hounsfield unit (HU) for diagnosing BMO with a sensitivity of 89% and a specificity of 90%.^56^ Diekhoff et al suggested the significant influence of reader experience on the diagnostic accuracy in their study, which involved readers with varying levels of training. They reported an average sensitivity of 72%, with the Fleiss’s kappa at 0.40.^57^ Overall, many studies have indicated the potential of DECT to replace MRI in acute settings in detecting BMO associated with vertebral fractures. The most recent meta-analysis, conducted by Sherbaf et al in 2021, reported a pooled sensitivity of 89% and specificity of 96%.^4^

### Knee injuries

**Figure 1.**
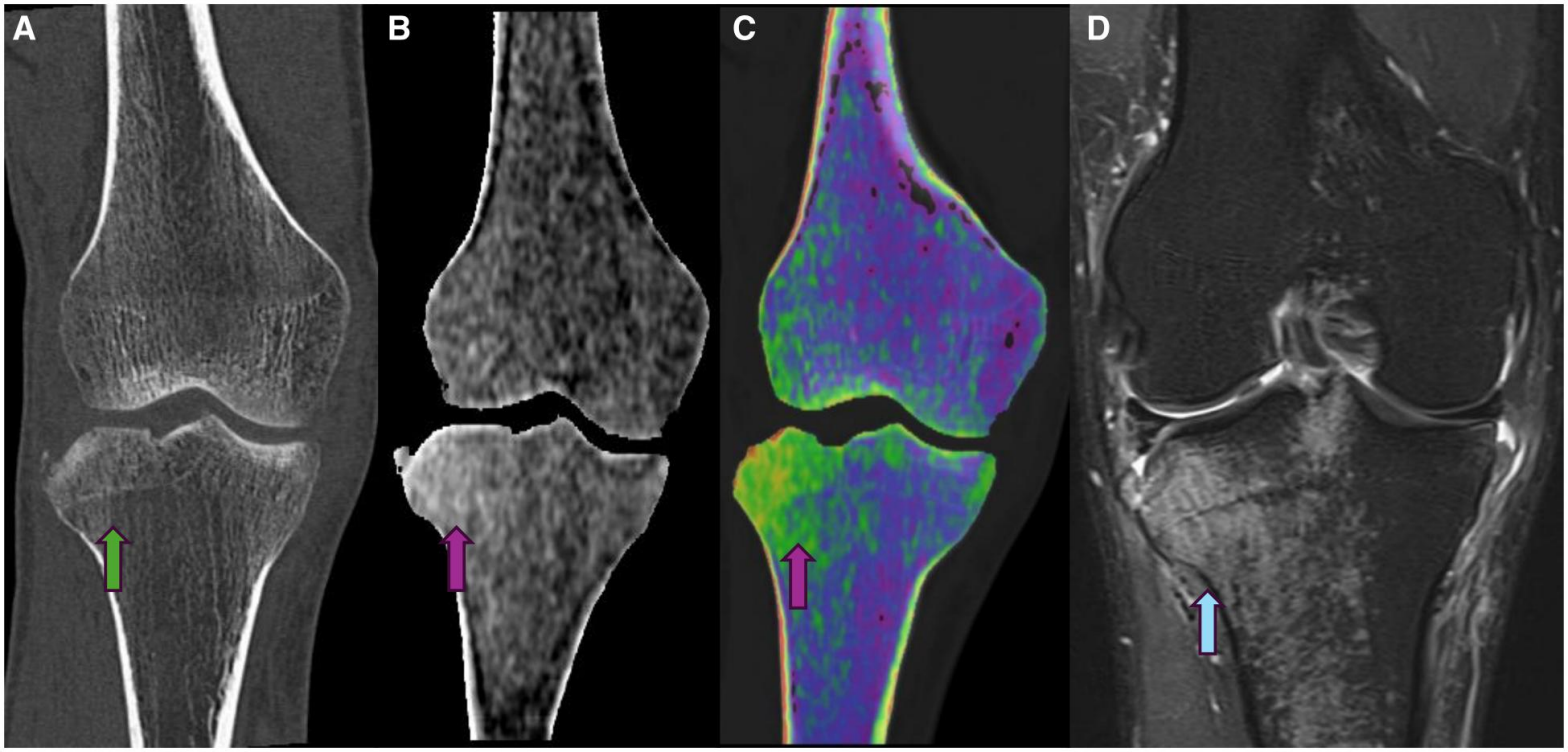
Patient with a history of acute knee injury trauma. **A** (green arrow) shows routine CT without significant difference of density and a Segond fracture. **B** and **C** show marrow oedema on DECT marrow oedema. Findings were confirmed with MRI as shown in **D.**

In their systematic review and meta-analysis, Li et al demonstrated the diagnostic capability of DECT in detecting traumatic knee injuries. Analysing 9 studies, they found the pooled sensitivity and specificity, using MRI as a reference standard, to be 85% (95% CI, 77%-90%) and 96% (95% CI, 93%-97%), respectively. In Ai et al’s^16^ study, where DECT images were obtained 11-99 days following the MRI images, they observed that DECT and MRI show good agreement up to 10 weeks. This is valuable information, as less urgent MRI imaging is often delayed, and DECT can provide insights where MRI scans are limited. Some of previous studies have also demonstrated a significant increase in the CT values in the oedematous knee joint regions. For example, in Wang et al’s study, they revealed 2 readers’ average CT values of −39.2 and −38.4 in oedematous regions as compared to −101.3 and −104.2 in non-oedematous regions (Figure 1).^17^

### Hip injuries

The majority of traumatic pelvic fractures are caused by low-energy impacts, including falls from stairs, which are commonly seen in the elderly population. One of the challenges in the diagnosis work-up is to identify occult fractures. Additionally, understanding the full scope of the fracture is equally important as the location of the fracture, and the number of fracture sites can greatly affect treatment decisions and prognosis.^58^ Palm et al showed that DECT was able to detect additional fractures compared to conventional CT and had a sensitivity of 100% using sacral insufficiency fracture (SIF)-associated BMO as a reference standard. DECT and MRI also yielded the same result in assessing the severity classification.^59^ Reddy et al examined 25 patients who had clinical suspicions of hip fracture but inconclusive radiographs. Among them, 21 patients were found to have BMO by DECT. Out of those, 3 patients were found to be false positives, as the BMO was ruled to be due to degenerative changes.^60^ In their study of 118 patients presenting to the emergency department with suspected nondisplaced hip fractures, Kellock et al^61^ reported comparable diagnostic confidences in the 22 positive cases using bone reconstructions alone versus using both bone reconstructions and VNCa images. However, they noted increased confidence when excluding fracture in the 96 negative cases when using both techniques.

### Wrist/scaphoid injuries

**Figure 2.**
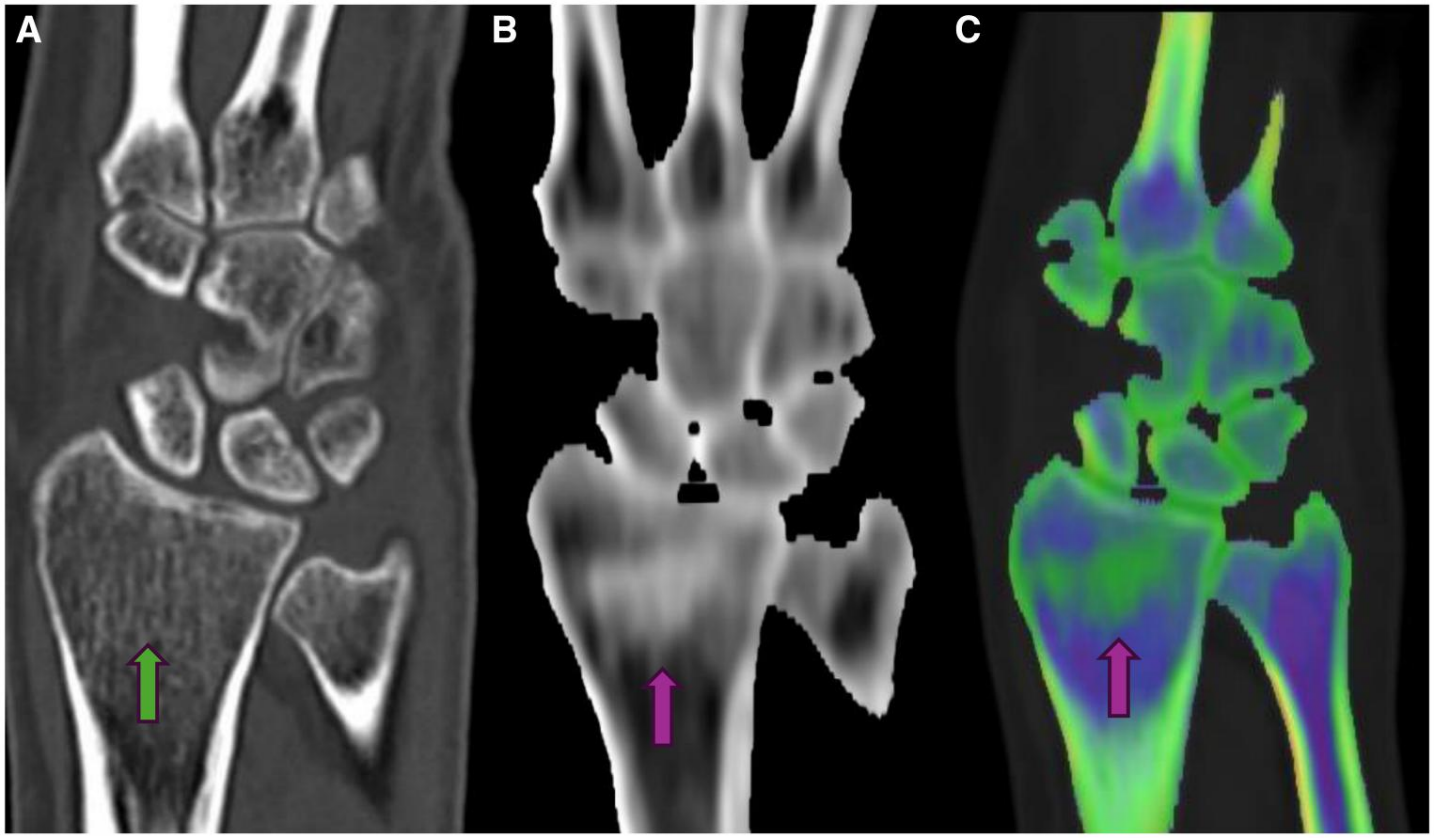
Patient with a history of acute wrist injury. **A** (green arrow): routine CT showing nonfracture or oedema. **B** and **C** show marrow oedema on DECT suggestive of a micro-trabecular fracture.

Scaphoid fracture is the most common carpal fracture, yet it poses a challenge for emergency physicians, as a certain number of occult fractures escape detection on initial radiographs.^62^ Xie et al conducted a direct comparison between DECT and MRI in the diagnosis of occult acute scaphoid injury; the 2 reviewers identified 10 and 13 cases of BMO compared to 14 detected by MRI.^63^ In the missed cases, the oedema was either mild or in the subcortical scaphoid region. In a separate study, Ali et al reported a cut-off value of 5.90 HU for detecting BMO associated with acute wrist fractures with sensitivity of 100% and specificity of 99.5% on DECT (80 and 140 kV) (Figure 2).^29^

### Ankle/foot injuries

DECT plays a significant role in the evaluation of foot and ankle injuries, particularly in the detection of BMO and subtle fractures that may not be visible on conventional CT images. DECT technology, with its advanced post-processing applications, allows for the generation of virtual calcium-suppressed images, which enables the visualisation of BMO through the subtraction of calcium from cancellous bone. This technique helps in identifying BMO, which serves as a marker for occult or subtle fractures that may not be visible on conventional CT images, thus aiding in the accurate diagnosis of acute foot and ankle trauma.^64^

DECT has shown high sensitivity and specificity in detecting BMO, making it a valuable tool for further investigating bone tissues when conventional radiographs are inconclusive. This technology is particularly useful in cases of radiographically occult nondisplaced fractures or soft tissue injuries. Guggenberger et al^64^ demonstrated in their study a high sensitivity (90%) and moderate specificity (80.5%) when employing Virtual non-calcium (VNCa) DECT for evaluating BMO as compared to MRI. Foti et al^65^ also found even more promising findings in a comparable study. In their investigation of acute ankle trauma using a third-generation DECT with VNCa algorithm, they achieved superior results, boasting both sensitivity and specificity of 100%.

Additionally, DECT enables a confident diagnosis, particularly in cases of small fractures, where the detection of BMO can pinpoint the location of a bone lesion. This early diagnosis can help decrease the need for further follow-up imaging, thereby lowering costs and minimising the patient’s cumulative radiation exposure.^66^

DECT can be integrated into the standard trauma imaging protocol in the Emergency Department, effectively tackling the issues of higher workload and the necessity for precise diagnosis of foot injuries.

### Ligament and tendon injuries

The role of DECT in ligamentous and tendon injuries is to provide a potential method for evaluating these soft tissue structures in the context of acute trauma.

DECT has been found to produce superior images when compared with conventional CT, likely due to the reduction of beam hardening artefacts, for example, within the phalanges of the finger spaces.^67^

DECT offers the ability to create material-specific colour mapping and dual-energy bone removal, which can aid in the visualisation and differentiation of ligaments and tendons from surrounding tissues. Additionally, DECT has the potential to provide higher soft tissue resolution compared to conventional polychromatic Multidetector computed tomography (MDCT) images, which may be beneficial for assessing injuries to these structures.

**Figure 3.**
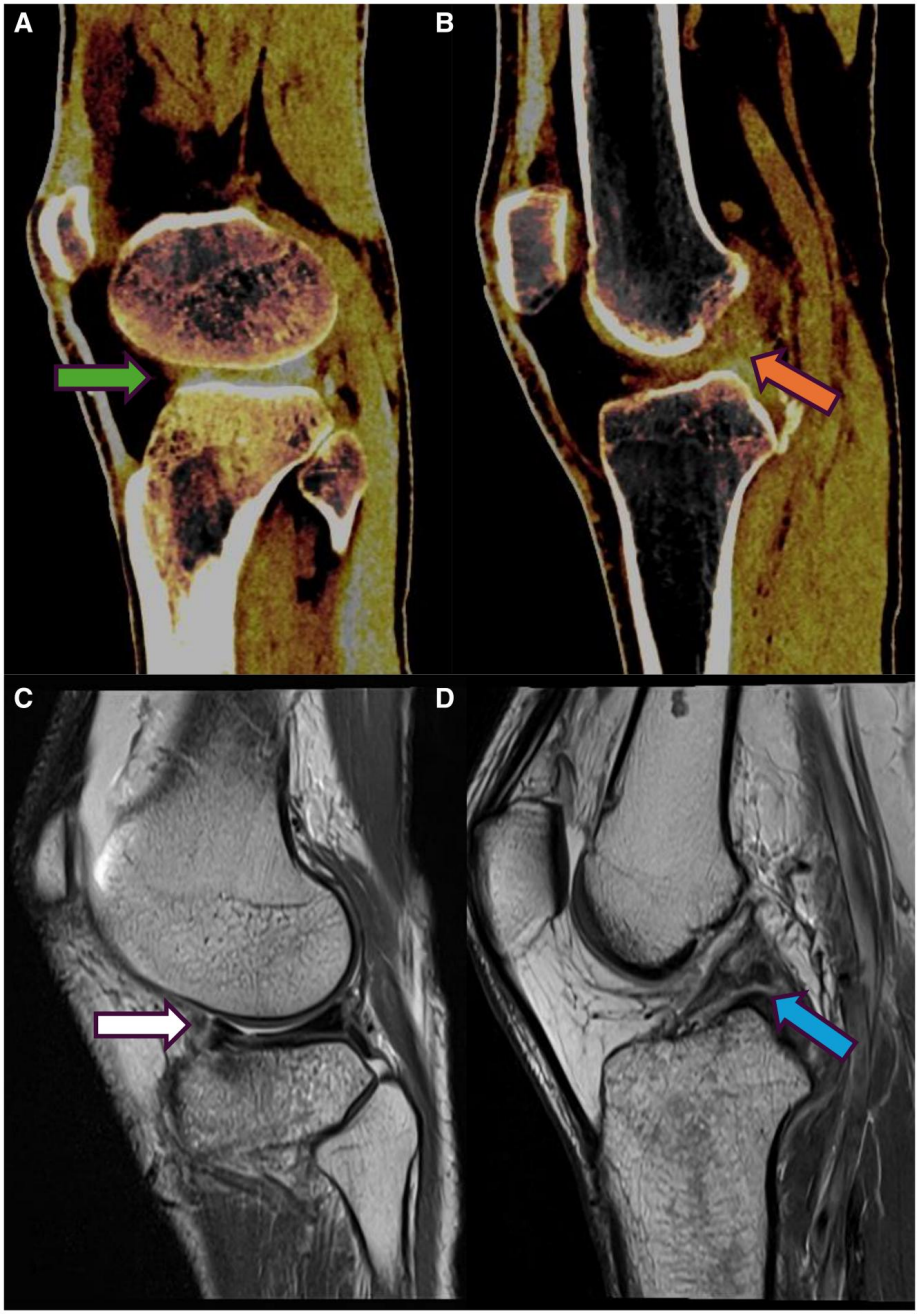
Patient with a history of acute trauma. **A** (green arrow): injury of the anterior horn and **B** shows injury of ACL as depicted on the DECT tendon map application. Findings were confirmed with MRI as shown in **C** and **D.**

In a study conducted at a level 1 trauma centre, DECT was used to evaluate cruciate ligament injuries in acute knee trauma patients. The study sought to determine the most effective keV level for DECT gemstone spectral imaging (GSI) and to evaluate the utility of collagen-specific colour mapping and dual-energy bone removal techniques in the assessment of cruciate ligaments and the popliteus tendon. The results of the study showed that DECT had a sensitivity of 79% and a specificity of 100% in detecting Anterior cruciate ligament (ACL) rupture, demonstrating its potential in the evaluation of ligamentous injuries (Figure 3).^68^

**Figure 4.**
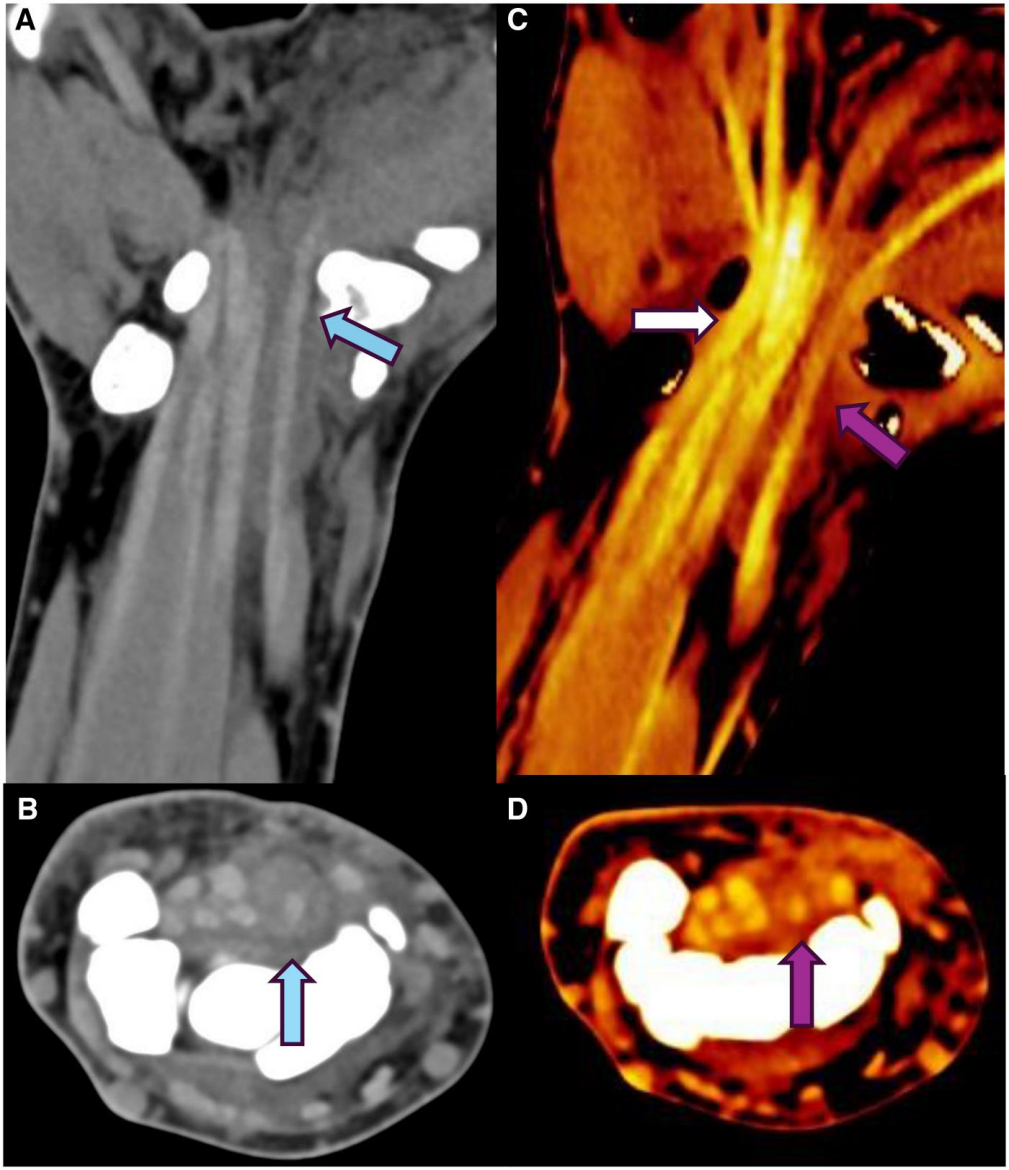
Patient with a history of acute wrist injury. **A** and **B** (blue arrow): routine CT without significant difference of density between the tendons. **C** and **D** show reduced density on dual energy tendon maps involving the flexor pollicis longus tendon in keeping with acute tendon injury as suspected clinically.

DECT has also demonstrated the ability to clearly image all tendons of the hand, as well as provide similar visualisation of the ACL compared to MR imaging (Figure 4).

In studies involving porcine hind legs, DECT and MR imaging showed similar ACL visualisation, as well as sensitivity and specificity for partial ACL tears. Additionally, DECT has been used to assess ligamentous injuries due to penetrating injuries of the wrist and ankle, providing adequate assessment of these injuries.^67^

## Challenges in detecting periprosthetic injuries

DECT offers several advantages in imaging patients with metallic implants. Metal artefact reduction (MAR) algorithms are used in DECT to reduce the impact of metal implants on CT images.^69^ This is particularly useful in cases where conventional radiography and MRI may be limited due to susceptibility artefacts from metallic implants.

**Figure 5.**
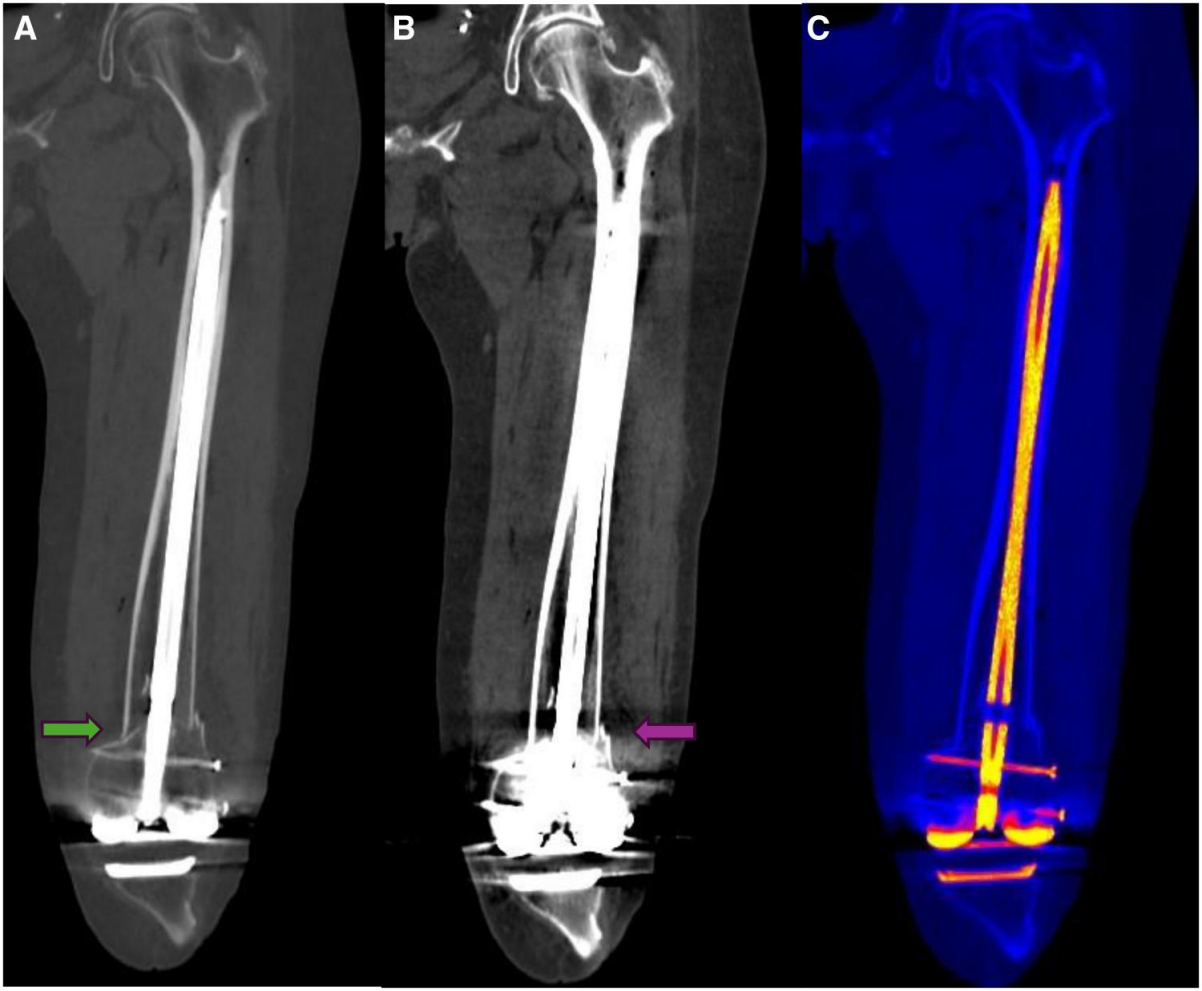
Patient with periprosthetic fracture after total knee replacement. **A** shows distal femoral fracture at high kV—140 with a reduced metallic artefact. The fracture of the medial aspect of the femur is seen as compared to **B** at low kV—60 with increased metal artefact. **C** shows the integrity of the hardware on dual energy maps for hardware.

Metal artefacts primarily arise from various mechanisms, including photon starvation, beam hardening, scattering, partial volume effects, undersampling, and patient motion. DECT addresses these challenges by utilising VMI. By adjusting the peak kilovolts settings (photon energy), DECT enables users to select the most suitable energy level, typically ranging from 40 to 140 keV, to achieve optimal diagnostic image quality (Figure 5).^70^

These algorithms work by implementing specific post-processing techniques for energy, which enables the creation of a virtual monochromatic energy spectrum. This helps in reducing the diagnostic quality of CT images caused by metal artefacts, allowing for a clearer study of the integrity of the implant, periprosthetic anatomical tissues, and relevant anatomical structures.^71^ These algorithms are especially valuable for post-surgical assessments in patients who have metal prostheses or osteosynthetic materials, enabling the evaluation of implant integrity, alignment, and the adjacent soft tissues for potential postoperative issues like fluid collections, haematomas, or periprosthetic fractures. Additionally, MAR algorithms have shown feasibility in reducing metallic dental artefacts, highlighting their potential value in maxillofacial traumas as well.^72^

Several studies have demonstrated the effectiveness of MAR algorithms in reducing metal artefacts in various clinical scenarios, such as spinal devices, total hip arthroplasty, and dental implants. DECT has been shown to enhance the reduction of beam hardening effects and improve diagnostic image utility.^73^^,^^74^

## Pitfalls and artefacts

DECT can present several artefacts that may affect the detection of marrow oedema. Some potential pitfalls of DECT in imaging BMO include the following^75–77^:

1. Photon Starvation and Beam Hardening: DECT may face challenges in imaging BMO due to photon starvation and beam hardening particularly in the presence in metal implants.^67^ This can lead to degraded image quality and inaccuracies in the visualisation of oedematous bone marrow.
2. Artefacts: DECT can produce artefacts, such as streak artefacts, which may affect the assessment of BMO. These artefacts can create false appearances of excessive or insufficient content, leading to potential diagnostic inaccuracies.
3. Limitations in Large Patients: DECT artefacts, particularly photon starvation, are more likely to occur in patients with larger body habitus, which can impact the visualisation and characterisation of BMO in this population.
4. Metal Artefact: Patients with metallic hardware may present challenges for DECT imaging, potentially affecting the accurate assessment of BMO in the presence of such artefacts.
5. Centring and Field of View: Poor patient centring within the gantry or inadequate inclusion of the area of interest within the dual-energy field of view can result in artefacts and limitations in assessing BMO.
6. Masking Effect: It can be challenging to assess BMLs adjacent to cortical bone due to the masking effect of the adjacent cortex. There is a loss of information due to spatial averaging, which removes high-attenuation pixels just beneath the cortex as part of the threshold-based segmentation in the VNCa algorithm of DECT, decreasing its sensitivity for such lesions.^67^

To address challenges in DECT imaging of BMO, several strategies can be employed. Firstly, educating CT technologists on the significance of precise patient centring and the inclusion of relevant anatomy within the dual-energy field of view is crucial. This awareness can help mitigate issues associated with inadequate positioning and coverage. Additionally, the utilisation of virtual monoenergetic images proves beneficial in reducing beam hardening artefacts, particularly at higher kiloelectronvolt (keV) levels, thereby enhancing image quality by minimising noise.

Furthermore, implementing MAR algorithms can significantly improve image quality by reducing artefacts caused by metallic hardware. These algorithms, applicable to both single-energy CT (SECT) and DECT images, offer notable enhancements, especially in areas susceptible to photon starvation effects. Spectral separation techniques, such as employing a tin filter in front of the high-energy source, are effective in overcoming photon starvation in patients with larger body size, thereby enhancing spectral separation and overall image quality.

Moreover, careful consideration of X-ray energy spectra, including the selection of appropriate kilovoltage peak (kVp) pairs and filters, can optimise spectral separation and minimise artefacts related to photon starvation and beam hardening. Radiologists should maintain awareness of potential artefacts, particularly in large patients and centrally located anatomy, during the interpretation of DECT post-processed images. This vigilance ensures accurate diagnosis and facilitates appropriate clinical decision-making. By integrating these strategies into practice, healthcare professionals can mitigate challenges associated with DECT imaging of BMO, ultimately improving patient care outcomes.^78^^,^^79^

## Conclusion

DECT represents a significant advancement in medical imaging, particularly for the detection and assessment of BMO. By leveraging the differential absorption properties of materials at 2 distinct X-ray energy levels, DECT offers enhanced tissue differentiation, providing critical diagnostic information that traditional imaging modalities may miss. This capability is particularly beneficial in the context of traumatic injuries, where early and accurate detection of BMO can significantly influence clinical management and treatment outcomes.

In addition to its diagnostic capabilities, DECT offers practical advantages such as reduced scanning time and immediate availability in emergency settings, making it a valuable tool for acute trauma assessment. Advanced post-processing techniques, such as material-specific colour mapping and virtual monochromatic imaging, further enhance the diagnostic utility of DECT by reducing artefacts and providing clear, detailed images.

Overall, DECT stands out as a powerful imaging modality that enhances the diagnostic landscape for BMO and related pathologies. Its integration into clinical practice, particularly in settings where MRI is not feasible, represents a significant step forward in the timely and accurate assessment of traumatic injuries and other conditions affecting the bone marrow. As technology continues to evolve, the role of DECT in medical imaging is poised to expand, offering even greater diagnostic precision and clinical utility.
